# miR‐373 regulates inflammatory cytokine‐mediated chondrocyte proliferation in osteoarthritis by targeting the P2X7 receptor

**DOI:** 10.1002/2211-5463.12345

**Published:** 2018-02-10

**Authors:** Wei Zhang, Biao Zhong, Chi Zhang, Congfeng Luo, Yulin Zhan

**Affiliations:** ^1^ Department of Orthopaedics Shanghai Jiao Tong University Affiliated Sixth People's Hospital Shanghai China

**Keywords:** chondrocyte, miR‐373, osteoarthritis, purinergic P2X7 receptor

## Abstract

Inflammatory cytokines commonly initiate extreme changes in the synovium and cartilage microenvironment of osteoarthritis (OA) patients, which subsequently cause cellular dysfunction, especially in chondrocytes. It has been reported that induction of the purinergic P2X7 receptor (P2X7R) can regulate the expression of a variety of inflammatory factors, including interleukin (IL)‐6 and ‐8, leading to OA pathogenesis. However, knowledge of the mechanism of upregulation of P2X7R in OA is still incomplete, and its role in chondrocyte proliferation is also not clear. It was reported previously that the expression of P2X7R was controlled by certain microRNAs, and so we tested the expression of several microRNAs and found that microRNA‐373 (miR‐373) was downregulated in the chondrocytes from OA patients. Regarding the mechanism of action, miR‐373 inhibited chondrocyte proliferation by suppressing the expression of P2X7R, as well as inflammatory factors such as IL‐6 and IL‐8. Furthermore, the proliferative and pro‐inflammatory effects of miR‐373 on the chondrocytes could be suppressed by a P2X7R antagonist, further suggesting that miR‐373 mediates chondrocyte proliferation and inflammation by targeting P2X7R. Generally, our results suggest a novel method for OA treatment by targeting the miR‐373–P2X7R pathway.

**Abbreviations**
BrdU: 5‐bromo‐2‐deoxyuridine
IL‐6/8: interleukin‐6/8
KL: Kellgren–Lawrence
miR‐373: microRNA‐373
MTT: 3‐(4,5‐dimethylthiazol‐2‐yl)‐2,5‐diphenyl‐tetrazolium bromide
OA: osteoarthritis
P2X7R: purinergic P2X7 receptor
RT‐PCR: real‐time PCR

As a kind of chronic degenerative disease, osteoarthritis (OA) is manifested clinically by a significant decrease in physical ability and an increase in morbidity with a high utilization of healthcare resources 1. The occurrence of OA involves many processes, including aging, proliferation and inflammation, which usually lead to an increased stress on some joints or cartilage, thereby resulting in cartilage damage 2, 3. It was generally thought that aging of chondrocytes, referred to chondrosenescence, is one of major risk factors for OA 4, 5. Other than chondrosenscence, the abnormal proliferation of chondrocytes, the resident cells of cartilage, also contributes to the progression of OA 6. Affected by a variety of inflammatory factors, including interleukin (IL)‐6 and IL‐8, the chondrocyte and cartilage microenvironment undergoes alterations in OA patients that subsequently contributes to cell dysfunction 7. Therefore, the chondrocytes’ status is considered as a key factor in determining cartilage degeneration.

The purinergic P2X7 receptor (P2X7R) acts as an important regulator in inflammation‐ and pain‐associated diseases, including OA 8. Recent evidence showed that P2X7R is involved in skeletal remodeling and mechanotransduction. In *P2X7R‐*knockout mice, not only was periosteal bone formation decreased in long bones without a difference in length, but also osteogenesis caused by mechanical loading was ameliorated 9, 10. Nevertheless, the mechanisms of action of P2X7R and its role in OA remain unclear. Recently, the MiRDB database predicted that miR‐373 would bind with the P2X7R 3′UTR and suppress the inflammation in OA 8, but further studies are required to discover their association and function in chondrocyte proliferation and inflammation.

To further study the relationship between miR‐373 and P2X7R, and their role in OA progression, we firstly examined the expression of several miRNAs in plasma and chondrocytes from 12 OA patients and 11 normal participants. Our results indicated that only miR‐373 was downregulated in the OA patients. The suppression of miR‐373 led to induction of P2X7R, which modulated chondrocyte proliferation and inflammatory cytokine expression. Furthermore, inhibition of P2X7R by its inhibitor abrogates the chondrocyte proliferation and cytokine expression modulated by a miR‐373 inhibitor (miRNAi), which might provide a novel strategy for OA treatment.

## Materials and methods

### Reagents

The oligonucleotides of miR‐373 precursor, inhibitor and miRNAs for negative control were purchased from BGI Tech (Shenzhen, China). P2X7R, control siRNAs and the hairpin‐it‐miRNA qPCR quantification kit were from Genepharma (Shanghai, China). The P2X7R antagonist (A438079), recombinant IL‐6 and IL‐8 were purchased from Abcam (Cambridge, MA, USA).

### Isolation of human chondrocytes and plasma collection

A total of 11 OA patients and 12 healthy participants for control were enrolled at Shanghai Jiao Tong University Affiliated Sixth People's Hospital for collection of chondrocytes and plasma. The clinical information for the participants is shown in Table 1, including the Kellgren–Lawrence (KL) score, the Osteoarthritis Research Society International (OARSI) grade and Kraus’ modified Mankin score. Samples from knee cartilage and blood of all participants were collected. The knee cartilage was placed in 0.25% trypsin–EDTA for digestion followed by centrifugation for isolating the chondrocytes. Serum plasma was isolated from blood by centrifugation at 1000 ***g*** for 10 min. Before study, all participants signed a written informed consent, and the study was approved by the Ethics Committee of Shanghai Jiao Tong University Affiliated Sixth People's Hospital (Ethics number: 2015‐79). After isolation, chondrocytes were cultured in Dulbecco's modified Eagle's medium (Shanghai Weike Biotechnology, Shanghai, China) containing glucose at a specific concentration, 1% penicillin and 10% fetal bovine serum (37 °C, 5% CO_2_ and an appropriate humidity).

**Table 1:** Clinical information of healthy participants and OA patients, including age, KL score, the OARSI grade and Kraus’ modified Mankin score

	Average age	KL score	OARSI grade	Kraus’ modified Mankin score
Healthy participants	42	0–1	1.8 ± 0.9	2.3 ± 0.5
Patients	49	2–4	17.9 ± 1.8	10.3 ± 1.2

### BrdU assay

After 16 h of culture, chondrocytes were stained with 5‐bromo‐2‐deoxyuridine (BrdU) for 8 h. We performed the BrdU incorporation assay to determine cell proliferation, and all operations were in accordance with the manufacturer's instructions (Roche Diagnostics GmbH, Mannheim, Germany). The absorbance at 450 nm was measured.

### MTT assay

To determine the viability of chondrocytes in wells, we added 10 μL 3‐(4,5‐dimethylthiazol‐2‐yl)‐2,5‐diphenyl‐tetrazolium bromide (MTT; Promega, Madison, WI, USA) at a concentration of 5 mg·mL^−1^ into medium for 2–4 h of incubation. After the purple precipitate was visible, the remaining culture medium in each well was replaced by 75 μL DMSO, and cells were incubated at room temperature in a dark place for 2 h. Thereafter, absorbance at a wavelength of 490 nm was measured.

### Real‐time PCR

Following the manufacturer's instructions, we isolated total RNA from samples using a TRIzol kit (Invitrogen, Carlsbad, CA, USA). One microliter of RNA was used for reverse transcription by a reverse transcription kit (Takara, Mountain View, CA, USA). PCR amplification was initiated in an ABI 7900HT Fast real‐time PCR (RT‐PCR) system (Thermo Fisher Scientific, Waltham, MA, USA) for measuring the mRNA expression of IL‐6, IL‐8, miR‐373 and P2X7R. Synthesis of all primers by RT‐PCR was completed by BGI Tech.

### Western blotting assay

In this experiment, proteins were isolated from chondrocytes. In short, cells were firstly rinsed in PBS, then placed in RIPA buffer for lysis, and centrifuged for collection of protein. Prior to SDS/PAGE, quantification of protein was performed using the BCA Protein Assay Kit (Thermo Fisher Scientific). Subsequently, a mixture of proteins was isolated via SDS/PAGE, and protein bands were transferred on a polyvinylidene fluoride membrane. Primary antibody of P2X7R (1 : 1000; Abcam), which was diluted in accordance with the experimental requirements, was added on the membrane for incubation at 4 °C for 24 h. Afterwards, the membrane was EDTA incubated with secondary antibody for 2 h. Finally, with an ECL kit, the bands on the membrane were visualized. In this procedure, protein expression of β‐actin (1 : 1000; Abcam) was used as the internal control.

### Cell transfection

After isolation of chondrocytes and culture in 96‐well plates, they were transfected with miR‐373 precursor, miRNAi and miRNA‐NC (negative control) using the Lipofectamine 2000 reagent (Invitrogen). The efficiency of transfection was detected by RT‐PCR.

### Statistical analysis

In this study, all data were expressed as the mean ± standard deviation, and spss 18.0 (SPSS Inc., Chicago, IL, USA) was used for data analysis. Statistical difference was determined by one‐way ANOVA, in which a *t* test was performed for intergroup comparison. *P <0.05 was taken as the level of significance.

## Results

### miR‐373 decreases in patients with OA

**Figure 1:**
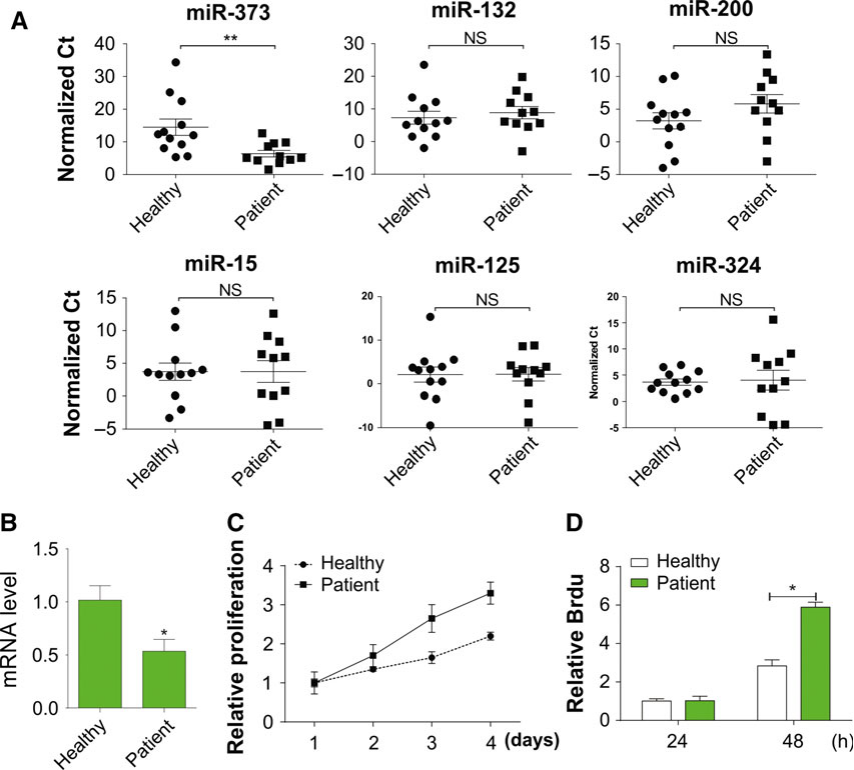
Expression of miR‐373 is suppressed in OA patients. (A) Analysis of error distribution in scatter plot of normalized Δ*C*_t_ values of miRNAs in plasma of healthy participants (*n* = 12) and OA patients (*n* = 11). (B) Analysis of miR‐373 expression in chondrocytes from healthy participants and OA patients by RT‐PCR. (C) Proliferation of chondrocytes in healthy participants and OA patients was determined by MTT assay at indicated time points. (D) Proliferation of chondrocytes from healthy participants and OA patients was determined by Brdu assay at indicated time points. Data are presented as mean ± SD from three independent experiments. *P < 0.05, **P < 0.01 compared with the control group.

To investigate the role of miRNA in OA, six miRNAs were randomly selected for investigation, namely miR‐373, miR‐132, miR‐200, miR‐15, miR‐125 and miR‐324. RT‐PCR was used to identify the expression levels of these six miRNAs with error distribution analyzed by their standard deviation. We found miR‐373 was significantly decreased in the circulating plasma of patients with OA compared with normal patients (Fig. 1A). To further confirm the regulation of miR‐373, we also analyzed its mRNA level in chondrocytes from OA patients and healthy participants, and found that chondrocytes from OA patient had lower miR‐373 expression (Fig. 1B). Further, chondrocytes derived from OA patients had a higher proliferation rate than those from healthy participants (Fig. 1C,D). The results demonstrated that inhibition of miR‐373 might stimulate chondrocyte proliferation and lead to OA.

### miR‐373 regulates chondrocyte proliferation

**Figure 2:**
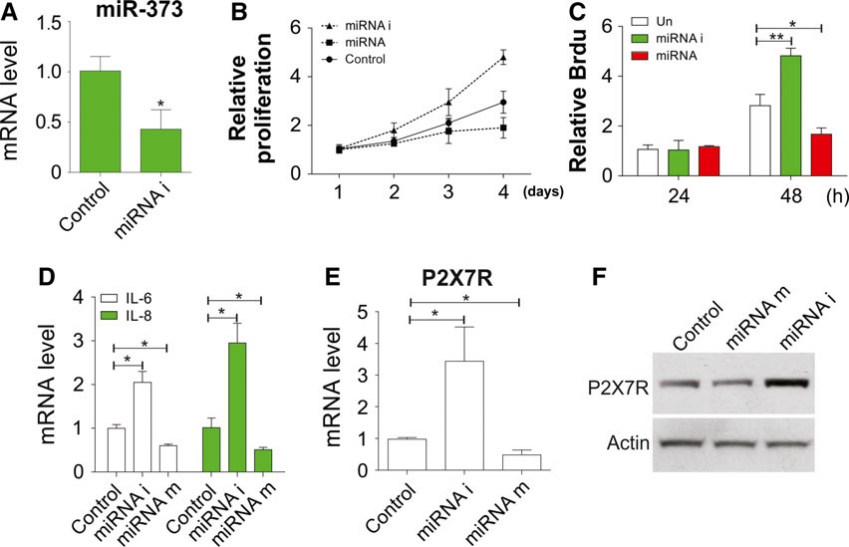
miR‐373 negatively regulates chondrocyte proliferation. (A) The suppression effect of miRNAi on its mRNA expression was determined by RT‐PCR. (B) Chondrocytes in OA patients were treated with inhibitor or precursor of miR‐373. The proliferation rate was determined by MTT assay at indicated time points. (C) Chondrocytes were treated as in (B) and the proliferation rate was determined by Brdu assay at indicated time points. (D) Chondrocytes were treated as in (B) and IL‐6 and IL‐8 expression was determined by RT‐PCR. (E) Chondrocytes were treated as in (B) and the expression of P2X7R was determined by RT‐PCR. (F) Chondrocytes were treated as in (B) and the expression of P2X7R was determined by western blot. Data are presented as mean ± SD from three independent experiments. *P < 0.05, **P < 0.01 compared with the control group.

As miR‐373 was downregulated in the plasma and chondrocytes of OA patients, we further assessed the functional role of miR‐373. To investigate how miR‐373 affects cell proliferation and cytokine secretion of chondrocytes, a specific oligonucleotides of inhibitor or precursor of miR‐373 was used for treatment, and we found that miR‐373 expression was altered. The RT‐PCR verified that the miRNAi actually repressed the expression of miR‐373 (Fig. 2A). As predicted, cell proliferation was promoted after miR‐373 expression was inhibited, as indicated by the results of MTT and Brdu assays (Fig. 2B,C). In contrast, the proliferation of chondrocytes treated by the miR‐373 precursor was significantly suppressed when compared with control chondrocytes (Fig. 2B,C). Accordingly, IL‐6 and IL‐8 were highly expressed in the presence of the miRNAi, whereas they were significantly suppressed by the miR‐373 precursor (Fig. 2D). The essential role of P2X7R in OA progression has been identified in previous studies 8. We also found that P2X7R mRNA and protein levels were reduced by the miR‐373 precursor, whereas they were increased by miR‐373 inhibition (Fig. 2E,F), which suggested that miR‐373 might reduce the P2X7R protein level to further affect chondrocyte proliferation and the concentrations of inflammatory factors.

### The expression of P2X7R is negatively correlated with miR‐373

**Figure 3:**
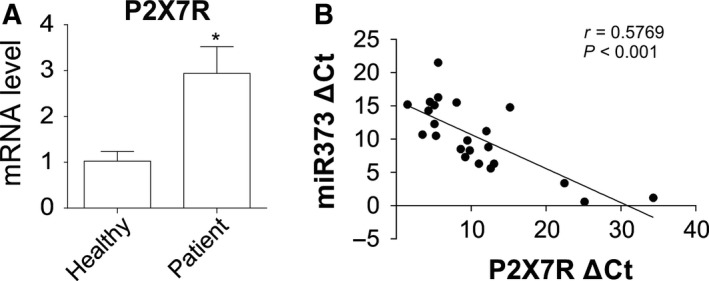
Expression level of P2X7R is negatively correlated with miR‐373. (A) P2X7R expression in chondrocytes of OA patients was determined by RT‐PCR, and normalized with that from healthy participants. (B) Correlation between P2X7R and miR‐373 was analyzed by Spearman's rank correlation analysis. Data are presented as mean ± SD from three independent experiments. *P < 0.05 compared with the control group.

To confirm the effect of P2X7R on progression of OA, we detected the mRNA expression of P2X7R in chondrocytes from OA patients and healthy participants, and the results showed a significant elevation in expression of P2X7R in chondrocytes of OA patients when compared with the control group (Fig. 3A). Moreover, miR‐373 was inversely correlated with P2X7R mRNA in OA tissues (Fig. 3B). These data suggested that miR‐373 suppressed OA by targeting P2X7R.

### P2X7R knockdown negatively regulates OA progression

**Figure 4:**
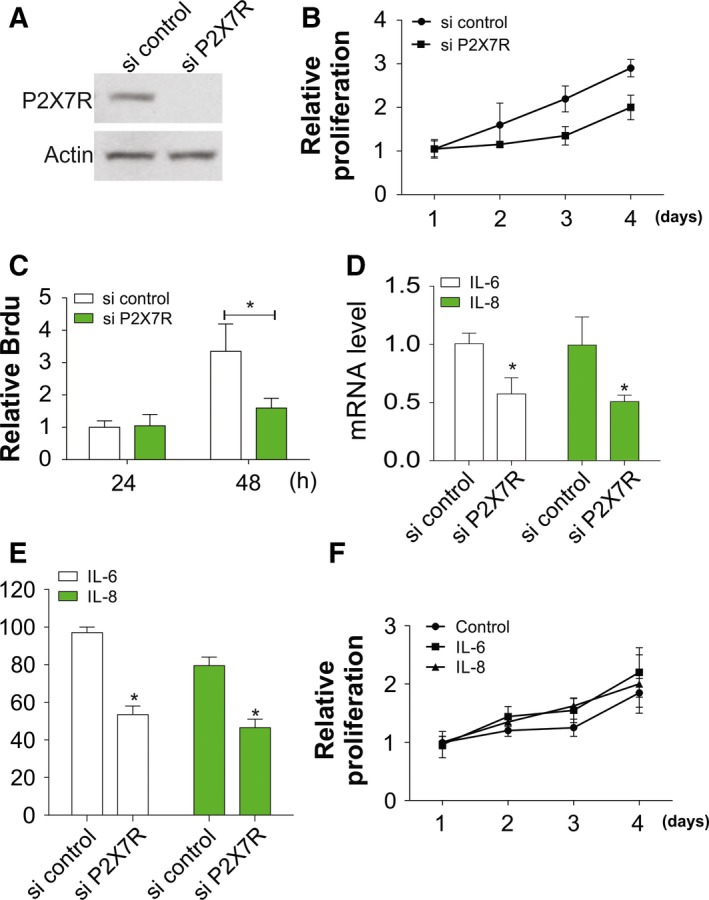
Knockdown of P2X7R inhibits the proliferation of chondrocytes. (A) Knockdown effect of P2X7R siRNA was verified by western blot. (B) Chondrocytes from OA patient were transfected with P2X7R siRNA. The proliferation rate was determined with an MTT assay at indicated time points. (C) Chondrocytes were treated as in (B), and the proliferation rate was determined by Brdu assay at indicated time points. (D) Chondrocytes were treated as in (B) and IL‐6 and IL‐8 expression as mRNA level was determined by RT‐PCR. (E) Chondrocytes were treated as in (B) and the secretion of IL‐6 and IL‐8 was determined by ELISA. (F) Chondrocytes from OA patient were transfected with P2X7R siRNA and treated with 100 ng·mL^−1^ IL‐6 or 100 ng·mL ^−1^ IL‐8. The proliferation rate was determined by MTT assay at indicated time points. Data are presented as mean ± SD from three independent experiments. *P < 0.05 compared with the control group.

As miR‐373 inversely regulated P2X7R expression, we tested whether P2X7R was involved in the proliferation and inflammation of chondrocytes. After P2X7R knockdown by siRNA, the cell proliferation and viability of chondrocytes were suppressed as exhibited by MTT and Brdu assays (Fig. 4A–C). Moreover, significant downregulation was identified in mRNA expression and secretion of IL‐6 and IL‐8 in chondrocytes (Fig. 4D,E). However, addition of IL‐6 or IL‐8 had no effect on cell proliferation, which was suppressed by P2X7R siRNA (Fig. 4F). These results indicated that P2X7R modulates chondrocyte proliferation, as well as controlling chondrocyte inflammation.

### P2X7R antagonist combats the effect of miR‐373 on chondrocytes

**Figure 5:**
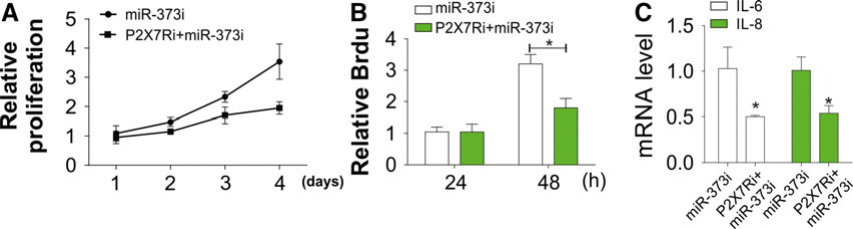
P2X7R antagonist reverses the inhibition effect of miRNAi on OA proliferation. (A) Chondrocytes of OA patients were treated with miRNAi with or without pretreatment with P2X7R antagonist. The proliferation rate was determined by MTT assay at indicated time points. (B) Chondrocytes were treated as in (A) and the proliferation rate was determined by Brdu assay at indicated time points. (C) Chondrocytes were treated as in (A) and IL‐6 and IL‐8 expression was determined by RT‐PCR. Data are presented as mean ± SD from three independent experiments. *P < 0.05 compared with the control group.

In this study, we determined whether P2X7R is involved in the inflammatory responses of chondrocytes mediated by miR‐373. Chondrocytes of healthy participants were pretreated with a P2X7R antagonist (A438079, 10 μM) for 1 h and then transfected with a miRNAi. The results showed that in chondrocytes, the effect of miR‐373 was counteracted by the P2X7R antagonist (Fig. 5A,B). Besides, the P2X7R antagonist also inhibited the upregulation of IL‐6 and IL‐8 caused by the miR373 inhibitor (Fig. 5C). The results revealed that the P2X7R antagonist protected chondrocytes against inflammation and proliferation mediated by miR‐373.

## Discussion

As a general type of arthritis, OA is affected by age, gender, history of joint injury, obesity, heredity and abnormal joint shape 2, 11. It is frequently described with clinical manifestations of cartilage destruction, subchondral bone sclerosis and osteophyte formation, which are caused by chronic and low‐grade inflammation 12, 13. In the development process of the skeleton, chondrocytes, originating from mesenchymal progenitors, are involved in synthesis of the templates or cartilage anlagen for bone development 14. Cartilage degradation can be promoted by chondrocytes through proliferation and secretion of inflammatory factors 6, 15. IL‐6 and IL‐8 are two important pro‐inflammatory cytokines that are potent regulators of chondrocyte functions 16. It was reported that increased concentrations of inflammatory factors, including IL‐6 and IL‐8, promote OA progression by increasing cartilage degradation 17. The overexpression of these two cytokines causes severe damage to chondrocyte function, resulting in an imbalance of cartilage homeostasis and finally articular degeneration 18. In our study, we also found that chondrocytes in OA patient have a higher proliferation rate, as well as expression of inflammatory cytokines, including IL‐6 and IL‐8. These results further suggest that suppressing the over‐proliferation of chondrocytes and specifically blocking the inflammation pathways could be a promising therapeutic treatment for OA.

As to the mechanism of chondrocyte proliferation, we found that the expression of miR‐373 is downregulated in OA patients, and this has a negative effect on regulation of chondrocyte proliferation. According to previous microarray and database analyses, a large number of miRNAs have been discovered in OA, in which some miRNAs (miR‐16, miR‐483, etc.) are upregulated in expression, while some (miR‐26a, miR‐337, etc.) are downregulated 19. Besides, miR‐373 can mediate the inflammation, thereby being involved in disease regulation. Coincident with the previous study, downregulation of miR‐373 expression was also found in OA chondrocytes in this study 8. In addition, the results of this study also revealed that the inhibition of miR‐373 promotes chondrocyte proliferation and secretion of inflammatory cytokines IL‐6 and IL‐8. However, enhanced miR‐373 has the reverse effects. These results indicated that miR‐373, as an OA inhibitor, is involved in the inflammation and functional variations. Additionally, miR‐373 can inhibit OA chondrocyte inflammation by targeting P2X7R, which is also consistent with previous reports 8.

The activity of P2X7R, an ionotropic receptor, is affected by ATP 20. P2X7R is widely expressed in a variety of tissues 21, including the musculoskeletal system; it has been postulated that the release of ATP in these tissues in response to mechanical loading can regulate their homeostasis 22. Expression of P2X7R also occurs in chondrocytes, and excessive mechanical stress on chondrocytes in OA may facilitate the release of ATP, which, in turn, activates the expression of P2X7R, thereby triggering the secretion of transglutaminase 2 23. In this study, the expression of *P2X7R*, as a potential target gene, was investigated to discover the effect of miR‐373 on OA chondrocytes, and we found that it was elevated in OA chondrocytes, which was modulated by miR‐373. The effects of P2X7R antagonists on inflammatory arthritis were assayed in some rodent models, with some success 24. Blocking the expression of P2X7R, particularly prior to the onset of disease, can significantly suppress synovial inflammation and alleviate damage to local tissues as well as mechanical hyperalgesia but without any effect on the systemic acute phase response. There are various ongoing confirmatory clinical studies, but no satisfactory results have been reported so far 25, 26. Moreover, the P2X7R antagonist could abolish the inflammatory effect of miR‐373, which provides a new therapeutic strategy for OA treatment.

## Conclusion

In summary, our study confirmed that suppression of miR‐373 results in chondrocyte proliferation and inflammation by inducing P2X7R expression. Inhibition or depletion of P2X7R could compromise the effects of miR‐373 on chondrocyte proliferation and inflammation. These results suggested that P2X7R and miR‐373 might be important targets for OA therapy. The upregulation of P2X7R or downregulation of miR‐373 has the potential to be a biomarker for OA detection. However, further work might be needed to figure out how P2X7R modulates the secretion of IL‐6, IL‐8 and other cytokines.

## Author contributions

WZ and CL conceived and designed the study. WZ, CL, CZ and YZ performed the experiments. CZ and BZ analysed the data. BZ and CL wrote the paper. WZ and YZ reviewed and edited the manuscript. All authors read and approved the manuscript.

## References

[1] Johnson VL and Hunter DJ (2014) The epidemiology of osteoarthritis. Best Pract Res Clin Rheumatol 28, 5–15. 24792942 10.1016/j.berh.2014.01.004

[2] Felson DT , Lawrence RC , Dieppe PA , Hirsch R , Helmick CG , Jordan JM , Kington RS , Lane NE , Nevitt MC , Zhang Y *et al* (2000) Osteoarthritis: new insights. Part 1: the disease and its risk factors. Ann Intern Med 133, 635–646. 11033593 10.7326/0003-4819-133-8-200010170-00016

[3] Mobasheri A , Matta C , Zakany R and Musumeci G (2015) Chondrosenescence: definition, hallmarks and potential role in the pathogenesis of osteoarthritis. Maturitas 80, 237–244. 25637957 10.1016/j.maturitas.2014.12.003

[4] Musumeci G , Szychlinska MA and Mobasheri A (2015) Age‐related degeneration of articular cartilage in the pathogenesis of osteoarthritis: molecular markers of senescent chondrocytes. Histol Histopathol 30, 1–12. 10.14670/HH-30.1 25010513

[5] Musumeci G , Castrogiovanni P , Trovato FM , Imbesi R , Giunta S , Szychlinska MA , Loreto C , Castorina S and Mobasheri A (2015) Physical activity ameliorates cartilage degeneration in a rat model of aging: a study on lubricin expression. Scand J Med Sci Sports 25, e222–e230. 25039883 10.1111/sms.12290

[6] Goldring MB (2012) Chondrogenesis, chondrocyte differentiation, and articular cartilage metabolism in health and osteoarthritis. Ther Adv Musculoskelet Dis 4, 269–285. 22859926 10.1177/1759720X12448454 PMC3403254

[7] Buckwalter JA , Mankin HJ and Grodzinsky AJ (2005) Articular cartilage and osteoarthritis. Instr Course Lect 54, 465–480. 15952258

[8] Jin R , Shen M , Yu L , Wang X and Lin X (2017) Adipose‐derived stem cells suppress inflammation induced by IL‐1beta through down‐regulation of P2X7R mediated by miR‐373 in chondrocytes of osteoarthritis. Mol Cell 40, 222–229. 10.14348/molcells.2017.2314 PMC5386960 28343378

[9] Ke HZ , Qi H , Weidema AF , Zhang Q , Panupinthu N , Crawford DT , Grasser WA , Paralkar VM , Li M , Audoly LP *et al* (2003) Deletion of the P2X7 nucleotide receptor reveals its regulatory roles in bone formation and resorption. Mol Endocrinol 17, 1356–1367. 12677010 10.1210/me.2003-0021

[10] Li J , Liu D , Ke HZ , Duncan RL and Turner CH (2005) The P2X7 nucleotide receptor mediates skeletal mechanotransduction. J Biol Chem 280, 42952–42959. 16269410 10.1074/jbc.M506415200

[11] Blagojevic M , Jinks C , Jeffery A and Jordan KP (2010) Risk factors for onset of osteoarthritis of the knee in older adults: a systematic review and meta‐analysis. Osteoarthritis Cartilage 18, 24–33. 19751691 10.1016/j.joca.2009.08.010

[12] Greene MA and Loeser RF (2015) Aging‐related inflammation in osteoarthritis. Osteoarthritis Cartilage 23, 1966–1971. 26521742 10.1016/j.joca.2015.01.008 PMC4630808

[13] Daghestani HN and Kraus VB (2015) Inflammatory biomarkers in osteoarthritis. Osteoarthritis Cartilage 23, 1890–1896. 26521734 10.1016/j.joca.2015.02.009 PMC4630669

[14] Goldring MB , Tsuchimochi K and Ijiri K (2006) The control of chondrogenesis. J Cell Biochem 97, 33–44. 16215986 10.1002/jcb.20652

[15] Komori T (2016) Cell death in chondrocytes, osteoblasts, and osteocytes. Int J Mol Sci 17, E2045. 27929439 10.3390/ijms17122045 PMC5187845

[16] Henrotin YE , De Groote DD , Labasse AH , Gaspar SE , Zheng SX , Geenen VG and Reginster JY (1996) Effects of exogenous IL‐1 beta, TNF alpha, IL‐6, IL‐8 and LIF on cytokine production by human articular chondrocytes. Osteoarthritis Cartilage 4, 163–173. 8895217 10.1016/s1063-4584(96)80012-4

[17] Xu L , Peng Q , Xuan W , Feng X , Kong X , Zhang M , Tan W , Xue M and Wang F (2016) Interleukin‐29 enhances synovial inflammation and cartilage degradation in osteoarthritis. Mediators Inflamm 2016, 9631510. 27433031 10.1155/2016/9631510 PMC4940582

[18] Gao SG , Li KH , Zeng KB , Tu M , Xu M and Lei GH (2010) Elevated osteopontin level of synovial fluid and articular cartilage is associated with disease severity in knee osteoarthritis patients. Osteoarthritis Cartilage 18, 82–87. 19747583 10.1016/j.joca.2009.07.009

[19] Tsezou A (2014) Osteoarthritis year in review 2014: genetics and genomics. Osteoarthritis Cartilage 22, 2017–2024. 25456297 10.1016/j.joca.2014.07.024

[20] Panupinthu N , Rogers JT , Zhao L , Solano‐Flores LP , Possmayer F , Sims SM and Dixon SJ (2008) P2X7 receptors on osteoblasts couple to production of lysophosphatidic acid: a signaling axis promoting osteogenesis. J Cell Biol 181, 859–871. 18519738 10.1083/jcb.200708037 PMC2396816

[21] Bartlett R , Stokes L and Sluyter R (2014) The P2X7 receptor channel: recent developments and the use of P2X7 antagonists in models of disease. Pharmacol Rev 66, 638–675. 24928329 10.1124/pr.113.008003

[22] Garcia M and Knight MM (2010) Cyclic loading opens hemichannels to release ATP as part of a chondrocyte mechanotransduction pathway. J Orthop Res 28, 510–515. 19890993 10.1002/jor.21025

[23] Aeschlimann D and Knauper V (2017) P2X7 receptor‐mediated TG2 externalization: a link to inflammatory arthritis? Amino Acids 49, 453–460. 27562793 10.1007/s00726-016-2319-8 PMC5332493

[24] McInnes IB , Cruwys S , Bowers K and Braddock M (2014) Targeting the P2X7 receptor in rheumatoid arthritis: biological rationale for P2X7 antagonism. Clin Exp Rheumatol 32, 878–882. 25288220

[25] Keystone EC , Wang MM , Layton M , Hollis S and McInnes IB (2012) Clinical evaluation of the efficacy of the P2X7 purinergic receptor antagonist AZD9056 on the signs and symptoms of rheumatoid arthritis in patients with active disease despite treatment with methotrexate or sulphasalazine. Ann Rheum Dis 71, 1630–1635. 22966146 10.1136/annrheumdis-2011-143578

[26] Stock TC , Bloom BJ , Wei N , Ishaq S , Park W , Wang X , Gupta P and Mebus CA (2012) Efficacy and safety of CE‐224,535, an antagonist of P2X7 receptor, in treatment of patients with rheumatoid arthritis inadequately controlled by methotrexate. J Rheumatol 39, 720–727. 22382341 10.3899/jrheum.110874

